# Concerns on the Effectiveness of Current COVID-19 Vaccines

**DOI:** 10.3389/fmicb.2022.848803

**Published:** 2022-06-06

**Authors:** Xingguang Li

**Affiliations:** ^1^Hwa Mei Hospital, University of Chinese Academy of Sciences, Ningbo, China; ^2^Ningbo Institute of Life and Health Industry, University of Chinese Academy of Sciences, Ningbo, China

**Keywords:** SARS-CoV-2, VOCs, VOIs, Omicron, COVID-19 vaccines

The outbreak of coronavirus disease 2019 (COVID-19), caused by severe acute respiratory syndrome coronavirus 2 (SARS-CoV-2), was first reported in Wuhan, China, in December 2019. As of 21 December 2021, a total of 275 648 933 confirmed cases, including 5 364 584 deaths in more than 200 countries, areas, and territories, have been reported globally by the Center for Systems Science and Engineering at Johns Hopkins University (https://www.arcgis.com/apps/dashboards/bda7594740fd40299423467b48e9ecf6) (Dong et al., 2020). The Omicron variant, designated by the World Health Organization (WHO) on 26 November 2021 (https://www.who.int/news/item/26-11-2021-classification-of-omicron-(b.1.1.529)-sars-cov-2-variant-of-concern), is the latest SARS-CoV-2 variant of concern (VOC) (Viana et al., 2021). Based on recent data (18 December 2021), Omicron has become the dominant VOC in the United States, accounting for 73.2% of all new COVID-19 cases according to the Centers for Disease Control and Prevention (CDC) (https://covid.cdc.gov/covid-data-tracker/#variant-proportions). Compared to previous SARS-CoV-2 variants of concern (VOCs) (i.e., Alpha, Beta, Gamma, and Delta) and variants of interest (VOIs) (i.e., Lambda and Mu), Omicron is a mutation-laden SARS-CoV-2 variant (https://www.who.int/en/activities/tracking-SARS-CoV-2-variants/), containing some 36 non-synonymous mutations in its spike protein (Hadfield et al., 2018) (https://covariants.org/variants/21K.Omicron#21L), several of which are thought to play key roles in ACE2 binding, transmissibility, and immune evasion (Cameroni et al., 2021) (Figure 1).

**Figure 1:**
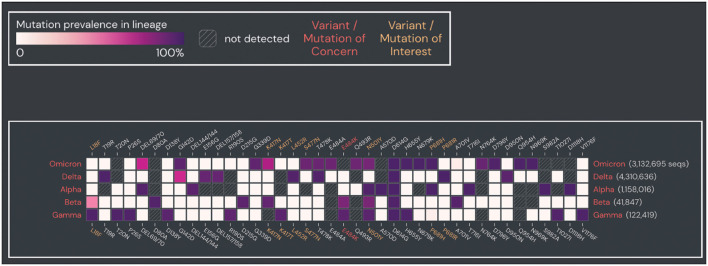
Prevalence of spike protein mutations in Omicron compared to other SARS-CoV-2 VOCs. Prevalence of spike protein mutations in Omicron compared to four SARS-CoV-2 VOCs (Alpha, Beta, Gamma, Delta). Mutations with >90% prevalence in at least one SARS-CoV-2 VOC and within >2 sequences are shown. Plot was generated from the outbreak website (https://outbreak.info/). Data were obtained from GISAID (as of 25 April 2022).

As of 21 December 2021, 19 127 genome sequences of Omicron from 78 countries have been submitted to GISAID (Elbe and Buckland-Merrett, 2017) EpiCoV (https://www.gisaid.org/hcov19-variants/). The estimated epidemic growth rate of Omicron in the United States is 0.23, corresponding to a doubling time of 3 days (Figgins and Bedford, 2021) (https://github.com/blab/rt-from-frequency-dynamics/tree/master/results/omicron-countries). The Omicron incubation period also appears to be shorter than that of Delta and other SARS-CoV-2 variants (Brandal et al., 2021; Grant et al., 2021), which may explain its rapid rise and fall in South Africa (https://ourworldindata.org/coronavirus/country/south-africa). Globally, as of 21 December 2021, 57% of people have received at least one dose of a COVID-19 vaccine, with 8.78 billion doses administered to date and 36.07 million doses currently administered each day (https://ourworldindata.org/covid-vaccinations). However, only 8.1% of people in low-income countries have received at least one dose, indicating large inequity in access to COVID-19 vaccines between high- and upper-middle-income countries and low-income countries. For instance, as of 20 December 2021, 56.95% of people worldwide have been vaccinated against COVID-19 (https://ourworldindata.org/covid-vaccinations), including 47.65 and 9.30% fully and partially vaccinated, respectively. Specifically, 72.7% of people in the United States have been vaccinated against COVID-19, including 61.0 and 11.7% fully and partially vaccinated, respectively, whereas, in Nigeria, only 4.16% of people have been vaccinated against COVID-19, including 1.96 and 2.20% fully and partially vaccinated, respectively.

Research indicates that Omicron infects and replicates 70 times faster than the original SARS-CoV-2 and Delta in human bronchi, which may indicate higher transmissibility (https://www.med.hku.hk/en/news/press/20211215-omicron-sars-cov-2-infection?utm_medium=social&utm_source=twitter&utm_campaign=press_release). However, Omicron also replicates more than 10 times lower in human lung tissue than the original SARS-CoV-2, this is consistent with an order of magnitude lower of titer of Omicron in lung tissue compared to Delta as recently reported (https://www.gla.ac.uk/media/Media_829360_smxx.pdf), which may be an indicator of less severe disease. This is consistent with research in South Africa showing a 70% (95% confidence interval: 50–80%) reduction in illness severity for Omicron compared to Delta (Wolter et al., 2021). This implies that Omicron may have undergone distribution drift, i.e., from cells in the lungs to cells in the bronchi, hinting that nasal vaccination may show better protection against Omicron than Delta or other SARS-CoV-2 variants (https://medicine.yale.edu/news-article/nasal-vaccines-may-protect-against-respiratory-viruses-better-than-injected-vaccines/). Recent study has suggested that Omicron is more transmissible (>three–five-fold) but less pathogenic than Delta (https://drive.google.com/file/d/1rhCazFav1pokFKmsZI5_oqIeH9ofFckR/view). Omicron also appears to show better immune evasion of neutralizing antibodies elicited from the adenovirus ChAdOx-1 vaccine than the mRNA BNT162b2 vaccine (Pfizer-BioNTech) (Meng et al., 2021). Furthermore, Omicron shows lower S1/S2 cleavage efficiency than the original SARS-CoV-2 and Delta variant, and less infectivity of Calu-3 lung cells than Delta (Meng et al., 2021). In addition, the fusogenicity of the Omicron spike protein shows impairment, resulting in less syncytium formation than Delta, consistent with recent research (https://drive.google.com/file/d/1rhCazFav1pokFKmsZI5_oqIeH9ofFckR/view). Thus, based on the abovementioned studies, Omicron shows reduced lung infectivity in comparison to Delta. Crucially, three independent studies in England (https://www.imperial.ac.uk/mrc-global-infectious-disease-analysis/covid-19/report-50-severity-omicron/), Scotland (https://www.research.ed.ac.uk/en/publications/severity-of-omicron-variant-of-concern-and-vaccine-effectiveness-), and South Africa (Wolter et al., 2021) also show a lower risk of hospital admission with Omicron infection.

Crucially, while neutralizing activity from mRNA double-vaccinated individuals is undetectable to very low against Omicron (Carreno et al., 2021), antibody neutralization is largely restored by a booster dose of mRNA vaccine (i.e., Moderna or Pfizer-BioNTech) (i.e., three doses of vaccine overall) (Carreno et al., 2021; Zeng et al., 2021). For example, 50-μg and 100-μg dose boosters of the Moderna vaccine can increase neutralizing antibody levels against Omicron by ~37-fold and 83-fold compared to pre-booster levels, respectively (https://investors.modernatx.com/news/news-details/2021/Moderna-Announces-Preliminary-Booster-Data-and-Updates-Strategy-to-Address-Omicron-Variant/default.aspx). Previous study showed that, against COVID-19-related hospitalization, effectiveness of two doses of Pfizer-BioNTech was 93% (95% confidence interval: 90–95) during 14–60 days but fell to 87% (95% confidence interval: 84–89) during 91–180 days, and effectiveness of three doses of Pfizer-BioNTech was increased to 96% (95% confidence interval: 95–97) during 14–60 days (https://www.researchsquare.com/article/rs-1489822/v1). Previous study showed that, against hospital admission, effectiveness of three doses of Pfizer-BioNTech against Omicron was 85% (95% confidence interval: 80–89) at <3 months but fell to 55% (95% confidence interval: 28–71) at 3 months or longer [https://www.thelancet.com/journals/lanres/article/PIIS2213-2600(22)00101-1/fulltext]. Previous study showed that, protection against severe COVID-19 and infection, effectiveness of four doses of Pfizer-BioNTech was 3.5-fold (unadjusted rate; 95% confidence interval: 2.7–4.6) and 2.0-fold (adjusted rate; 95% confidence interval: 1.9–2.1) lower rates than three doses of Pfizer-BioNTech, respectively (Bar-On et al., 2022). Thus, there are several strategies to increase vaccine effectiveness, including increasing the number of inoculations, increasing the dose of vaccinations, and combining different vaccines (e.g., different vaccines for first, second, and booster doses). However, these strategies need to be urgently evaluated.

Research has shown that the Johnson & Johnson vaccine is less effective than the mRNA vaccines (i.e., Moderna and Pfizer-BioNTech), and thus provides poorer protection (Garcia-Beltran et al., 2021). Another study has shown that two doses of COVID-19 vaccine are unlikely protective against Omicron, and the third dose of an mRNA vaccine provides substantially less protection against Omicron (37%, 95% confidence interval, 19–50) than Delta (93%, 95% confidence interval, 92–94) (Buchan et al., 2022). Therefore, based on concern regarding current vaccine effectiveness, I, a virologist in Ningbo, Zhejiang Province, China, strongly recommend that people, at this stage, vaccinate with mRNA vaccines (for instance, Moderna or Pfizer-BioNTech) for their first dose (unvaccinated people), second dose (partially vaccinated people), and booster dose (fully vaccinated people) prior to the emergence of universal COVID-19 vaccines (Morens et al., 2021; Li, 2022).

As such, it is important for the WHO to evaluate the effectiveness of the various mRNA- or non-mRNA-based vaccines against novel SARS-CoV-2 VOCs and VOIs among people of all ages (e.g., efficacy of vaccines and boosters, optimal doses of each vaccine, and safety of vaccine combinations). Although COVID-19 non-mRNA vaccines (e.g., Johnson & Johnson vaccine) can be used for partial and full vaccination, booster doses appear to be less effective against novel SARS-CoV-2 VOCs and VOIs compared to the mRNA vaccines. Notably, a previous study showed that Omicron formed a novel antigenic cluster associated with immune escape compared to the preceding four VOCs (Alpha, Beta, Gamma, and Delta) of SARS-COV-2 (van der Straten et al., 2022), indicating an urgent need for updated vaccines (Morens et al., 2021; Li, 2022). The growing COVID-19 vaccine hesitancy seen in developing countries is likely related to people wanting a much more effective one-dose vaccine that can protect over an entire lifetime (Li, 2022), rather than soon-to-expire “leftover” vaccines from high- and middle-income countries that require many doses over a short period of time and are less effective. Therefore, I strongly recommend that the WHO formally evaluate the effectiveness of each COVID-19 vaccine to allow people to make an informed choice on which vaccine to take, rather than being subjected to an endless cycle of boosters, which could waste medical and financial resources and exacerbate vaccine inequity.

## Author Contributions

XL conceived and designed the study and drafted the manuscript.

## Conflict of Interest

The author declares that the research was conducted in the absence of any commercial or financial relationships that could be construed as a potential conflict ofinterest.

## Publisher's Note

All claims expressed in this article are solely those of the authors and do not necessarily represent those of their affiliated organizations, or those of the publisher, the editors and the reviewers. Any product that may be evaluated in this article, or claim that may be made by its manufacturer, is not guaranteed or endorsed by the publisher.
